# Connexin50 hemichannels are opened by CO_2_: implications for lens physiology

**DOI:** 10.3389/fphys.2026.1869751

**Published:** 2026-07-22

**Authors:** Alexandra Lovatt, Frederick Bibra, Ashvini Wijayapala, Macy Mui, Jack Butler, Nicholas Dale

**Affiliations:** School of Life Sciences, University of Warwick, Coventry, United Kingdom

**Keywords:** CO_2_, connexin, hemichannel, lens fiber cells, microcirculation

## Abstract

Connexin50 (Cx50) is expressed in lens fiber cells. As mutations in Cx50 cause cataracts, its physiological role in the lens must be important. We have used recent cryo-EM structures of Cx50 and the predictive power of Alphafold3 to identify the presence of a carbamylation motif, originally described in Cx26, that suggests that Cx50 might be CO_2_ sensitive. By expressing the full-length and a naturally C-terminal truncated version of Cx50 in HeLa cells and utilizing coexpression of the genetically encoded sensors iGluSnFr or eLACCO1.1, we have demonstrated the CO_2_-dependent opening of both full-length and truncated Cx50 hemichannels. By mutating the two key residues of the carbamylation motif, K105 and K140, in the truncated version of Cx50, we have shown that the motif is required for the CO_2_ sensitivity of Cx50. Mutations of residue V44 cause cataracts, and these mutations abolish the CO_2_ sensitivity of Cx50. Using Fluo-4 Ca^2+^ imaging with lens slices, we have demonstrated CO_2_-dependent Ca^2+^ influxes into fiber cells that are blocked by La^3+^ and exhibit the same CO_2_ dose dependence as Cx50 hemichannels. Lens fiber cells respond to glutamate via NMDA receptors, and our data show that the Ca^2+^ influx in response to raised PCO_2_ partially depends on NMDA receptor activation. We hypothesize that CO_2_-dependent gating of Cx50, the subsequent release of glutamate resulting in the downstream activation of glutamate receptors, and the consequent alterations in transmembrane Na^+^ fluxes provide homeostatic control of the microcirculation system that is critical for lens health.

## Highlights:

- Cx50 hemichannels are opened by small changes in PCO_2_ via a carbamylation mechanism similar to that described for Cx26 and Cx43 and will be partially open at the PCO_2_ of the aqueous humor.
- Lens fiber cells dye load in a CO_2_-dependent manner and exhibit CO_2_-evoked Ca^2+^ influxes that are hemichannel mediated.
- Glutamate evokes Ca^2+^ influx into lens fiber cells via activation of NMDA receptors.
- The CO_2_-evoked Ca^2+^ influx into lens fiber cells is partially mediated via NMDA receptors activated by glutamate released via Cx50 hemichannels.
- We propose that Cx50 hemichannels mediate homeostatic control of lens microcirculation.

## Introduction

Connexin50 (Cx50) is an alpha connexin that shares considerable sequence homology and structural similarity with Cx46 (Myers et al., 2018; Flores et al., 2020). Both Cx50 and Cx46 are part of the alpha connexin clade, which also includes Cx43 (Cruciani and Mikalsen, 2006). These three connexins are all expressed in the mammalian lens (Mathias et al., 2010; Berthoud and Ngezahayo, 2017). While Cx43 is found only in the lens epithelium, Cx46 and Cx50 form gap junctions between lens epithelial and lens fiber cells and between lens fiber cells. Both Cx46 and Cx50 are also extensively expressed across the surface of lens fiber cells in the form of hemichannels (Lo et al., 1996; Beyer and Berthoud, 2014; Berthoud and Ngezahayo, 2017; Shi et al., 2018; Wang et al., 2025)—unopposed hexameric channels that open into the extracellular space.

To maintain transparency, the lens does not have a blood supply (Mathias et al., 1997). Essential nutrients are supplied, and metabolic waste products are removed, via a microcirculation system (Mathias et al., 1997, 2007; Delamere and Tamiya, 2009). This is powered by Na^+^ extrusion from equatorial epithelial cells. The movement of Na^+^ ions through the gap junction-coupled network of lens cells drags along water and other solutes. Water and these solutes re-enter the lens at the poles through the expanded extracellular space provided by the lens sutures.

An open question remains as to the pathway by which Na^+^ ions enter lens fiber cells (Mathias et al., 1997) to drive extrusion via the Na^+^/K^+^ pumps in the epithelial cells and thus the microcirculation. Cx50 has been discounted as the source because the hemichannels are deemed to be closed, and instead an unspecified leak channel has been proposed (Mathias et al., 2007). Hemichannels of Cx46 have been proposed to contribute to this leak current (Ebihara et al., 2014). In this study, we show that Cx50 hemichannels are directly sensitive to gaseous CO_2_, which acts via a binding mechanism and motif that we have previously described in Cx26 (Meigh et al., 2013). In Cx26, CO_2_ forms a covalent carbamate bond with Lys125 (Nijjar et al., 2025). The subsequent carbamate is negatively charged and can interact with Arg104 of the neighboring subunit. The carbamate bridges are thought to trap the Cx26 hemichannel in the open configuration (Brotherton et al., 2022, 2024). Cx26 is not unique in its CO_2_ sensitivity; hemichannels of Cx30 (Huckstepp et al., 2010a), Cx32 (Huckstepp et al., 2010a; Dospinescu et al., 2019; Butler and Dale, 2023), and, most recently, Cx43 (Dospinescu et al., 2026) are also opened by CO_2_ acting via equivalent motifs.

The properties of Cx50 hemichannels indicate that they will be partially open at PCO_2_ levels typical of the aqueous humor (Krupin et al., 1980). This suggests that Cx50 hemichannels have the correct properties to be the source of the Na^+^ leak current that underlies the microcirculation. By using Ca^2+^ imaging with acute lens slices and varying PCO_2_, we have provided some evidence that supports this hypothesis.

## Materials and methods

### Connexin mutagenesis

A cDNA sequence for the human Cx50 gene (accession P48165) truncated to 277 amino acids, was synthesized by IDT. This truncation of the human gene is equivalent to the natural age-related truncation at His284 documented for the bovine gene (Wang and Schey, 2009). While most of our anaysis was performed on the truncated gene, as there are many truncation variants of Cx50, we also obtained the full-length human gene to verify that the full-length proyein was also sensitive to CO_2_.

The cDNA sequences were subsequently subcloned into the pCAG-GS-mCherry vector prior to transfection. Point mutations were introduced using Gibson assembly. Overlapping primer fragments (IDT), both containing the desired mutation, were amplified by PCR. Successful mutagenesis was confirmed using Sanger sequencing (GATC Biotech). All Cx50 constructs were inserted upstream of an mCherry tag and linked via a 12-aa linker (GVPRARDPPVAT).

### Cell culture and transfection

Parental HeLa DH cells (ECACC 96112022, RRID: CVCL_2483) were cultured in low-glucose DMEM *(*Merck Life Sciences UK Ltd, CAT# D6046) supplemented with 10% fetal bovine serum (Labtech.com, CAT# FCS-SA) and 5% penicillin/streptomycin. Cells were seeded onto coverslips at a density of 4 × 10^4^ cells per well. Cells were transiently transfected to co-express a Cx50 variant and one of the following genetically encoded fluorescent sensors:

pCMV(MinDis).iGluSnFR was a gift from Loren Looger (Addgene plasmid #41732; http://n2t.net/addgene:41732; RRID: Addgene_41732) (Marvin et al., 2013).

pAEMXT-eLACCO1.1 was a gift from Robert Campbell (Addgene plasmid #167946; http://n2t.net/addgene:167946; RRID: Addgene_167946) (Nasu et al., 2021). To improve expression of eLACCO1.1, this construct was subcloned into the iGluSnFR expression vector backbone. Sequences were verified with Sanger sequencing (GATC).

To transfect cells, a mixture of 1 μg of DNA from the pCAG-Cx-mCherry construct, 1 μg of sensor DNA, and 3 μg of PEI was added to the cells for 4 h–8 h. Cells were imaged 48 h after transfection.

### Recording solutions used

20 mmHg PCO_2_: 140 mM NaCl, 10 mM NaHCO_3_, 1.25 mM NaH_2_PO_4_, 3 mM KCl, 1 mM MgSO_4_.
35 mmHg PCO_2_: 124 mM NaCl, 26 mM NaHCO_3_, 1.25 mM NaH_2_PO_4_, 3 mM KCl, 1 mM MgSO_4_.
55 mmHg PCO_2_: 100 mM NaCl, 50 mM NaHCO_3_, 1.25 mM NaH_2_PO_4_, 3 mM KCl, 1 mM MgSO_4_.
70 mmHg PCO_2_: 70 mM NaCl, 80 mM NaHCO_3_, 1.25 mM NaH_2_PO_4_, 3 mM KCl, 1 mM MgSO_4_.

High K^+^ (20 mmHg PCO_2_): 93 mM NaCl, 10 mM NaHCO_3_, 1.25 mM NaH_2_PO_4_, 50 mM KCl, 1 mM MgSO_4_.10 mM D-glucose and 2 mM CaCl_2_ were added to all solutions just before use. Solutions were saturated with 98%O_2_/2% CO_2_ (20 mmHg); 95% O_2_/5% CO_2_ (carbogen) (35 mmHg), or carbogen plus additional CO_2_ (55 mmHg and 70 mmHg). The amounts of CO_2_ were adjusted so that all solutions had a pH of ~7.4.

### Live cell fluorescence imaging and analysis

Forty-eight hours after transfection, cells were perfused with control aCSF (20 mmHg) until a stable baseline was reached before switching to either hypercapnic or high K^+^ aCSF. Once a stable baseline was reached after the solution change, cells were returned to perfusion with control aCSF (20 mmHg). Recordings were calibrated by applying 3 μM of the corresponding analyte.

All cells were imaged by epifluorescence (Scientifica Slice Scope, Cairn Research OptoLED illumination, Olympus ×60 water immersion objective, NA 1.0, Hamamatsu ImagEM EM-CCD camera, Metafluor software). cpGFP in the sensors was excited by a 470 nm LED, with emission captured between 504 nm and 543 nm. Connexin constructs carried a C-terminal mCherry tag, which was excited by a 535 nm LED, and emission captured between 570 nm and 640 nm. Only cells expressing both cpGFP and mCherry were selected for recording, with cpGFP images acquired every 4 s. For each condition, at least three independent transfections were performed with at least two coverslips per transfection.

Analysis of all experiments was carried out in ImageJ. Images were opened as a stack and stabilized to correct for movement (Li, 2008). ROIs were drawn around cells co-expressing both the sensor and connexin. Median pixel intensity was plotted as normalized fluorescence change (ΔF/F_0_) over time to generate fluorescence traces. The amount of analyte release was quantified as a concentration by normalizing to the ΔF/F_0_ produced by application of 3 μM analyte, which was within the linear portion of the dose–response curve for each sensor.

### Membrane localization of Cx50 and mutants

HeLa cells adhered to coverslips were transfected with mCherry-tagged wild-type and mutant versions of Cx50. After 48 h, the cells were washed three times with PBS and fixed with 4% paraformaldehyde in PBS for 30 min. They were then washed three times with PBS and incubated with serum-free DMEM containing 2.5 μM DiO (3,3′-Dioctadecyloxacarbocyanine perchlorate, Sigma-Aldrich Cat#D4292) for 15 min. After three further washes in PBS, coverslips were mounted on glass microscope slides using Fluorshield™ with DAPI mounting medium (Sigma-Aldrich, Cat# F6057). They were then imaged on a Zeiss 980 LSM confocal microscope using the 488 nm and 561 nm excitation wavelengths for DiO and mCherry, respectively.

Colocalization analysis between the mCherry tag of the Cx50 variants and the DiO membrane stain was performed using Fiji and the JaCoP plugin (Bolte and Cordelieres, 2006). ROIs were drawn around mCherry-positive cells, and the image surrounding each ROI was removed. The Manders’ co-efficient (Manders et al., 1993) was used as a quantitative measure of colocalization and, hence, the membrane localization of the Cx50 variants. The images were thresholded to include DiO membrane staining while excluding diffuse background fluorescence. Analysis was performed on images from a single optical plane.

### Dye loading of whole lens

Mice of either sex were euthanized by cervical dislocation in accordance with the United Kingdom Animals (Scientific Procedures) Act 1986. The eyes were isolated, and the intact lens was harvested and immediately perfused with control (20 mmHg) aCSF. For the experiments, samples were transferred to either control aCSF, hypercapnic aCSF, or hypercapnic aCSF containing 200 µM LaCl_3_ (Sigma-Aldrich, Cat #10025-84-0), with each solution containing 50 µM fluorescein isothiocyanate (FITC) (Sigma-Aldrich Cat #46950) for 10 min. Samples were then perfused with FITC-containing control aCSF for 20 min to prevent dye leakage.

Lenses were then washed sequentially in control aCSF and fixed in 4% paraformaldehyde (PFA) for 2 h at room temperature. Fixed tissue was washed at room temperature in PBS. The tissue was then embedded in 4% PBS-based agarose gel and mounted in an ice-laden sectioning chamber. Slices were cut either coronally or transversely to a thickness of 105 µm using a vibratome (Leica VT1200), and arranged sequentially in 24-well plates containing PBS. Free-floating sections were then treated with 20 µM DiI (Sigma-Aldrich, Cat #468495), a lipophilic membrane stain, for 30 min at room temperature. These samples were washed, mounted on poly-L-lysine-coated slides (Polysine, VWR), and dehydrated at room temperature for 10 min. Coverslips were applied using FluorshieldTM with DAPI mounting medium (Sigma-Aldrich, Cat #F6057). Specimens were imaged with a Zeiss 980 confocal microscope using a ×63 oil-immersion objective, and the 488- and 561-nm lasers, with the same imaging settings used for all slices to allow direct comparison of fluoresence intensity.

Images were again analyzed in FIJI, with the analysis performed blind to the perfusion condition. ROI’s were drawn around individual cells to measure mean pixel intensity and, thus, the extent of dye loading. Measurements were calibrated by subtracting the mean pixel intensity of a representative background ROI within the same image. A minimum of five lenses were included per condition, with the mean fluorescence intensity from at least 10 cells used as the data point.

### Preparation of lens slices

Live whole lenses were incubated for 18 min in the decapsulation solution first described by Dewey et al. (1995): 149.2 mM KMeSO_3_, 5 mM HEPES, 2 mM EGTA, 2 mM EDTA, 5 mM glucose, and 0.10 mM bumetanide (diluted from a 10 mM stock solution in absolute ethanol). This treatment allowed separation of the capsule with minimal teasing.

Lenses were individually embedded in 10% ultra-low-gelling-temperature agarose (Sigma-Aldrich, Cat # A5030) prepared in 20 mmHg Ca^2+^-free aCSF, placed in an ice-laden cutting chamber, submerged in 20 mmHg Ca^2+^-free aCSF containing 1 mM kynurenic acid, and sectioned either coronally or transversely using a vibratome to obtain a single central slice 300 µm thick.

### Ca^2+^ imaging of lens slices

Fluo-4-AM (Invitrogen, Cat #F14201), dissolved in Pluronic™ F-127 (Invitrogen, Cat #P3000MP) with the aid of sonication and vortexing, was diluted in 20 mmHg aCSF to a final concentration of 2.5 μM. Lens slices were incubated for 25 min in a custom humidified perfusion microchamber, with superperfusion of 98% O_2_/2% CO_2_ to maintain pH and PO_2_, before a subsequent 25 min wash with 20 mmHg aCSF.

Loaded lens slices were placed in a perfusion chamber and subjected to control aCSF until a stable baseline was maintained. Changes in Fluo-4 fluorescence intensity from baseline, defined as ΔF/F_0_, were measured by epifluorescence (Scientifica Slice Scope, Cairn Research OptoLED illumination, 60x water Olympus immersion objective, NA 1.0, Hamamatsu ImagEM EM-SSC camera, Optofluor software). Fluo-4 was excited using a 470 nm LED, with fluorescence emission recorded between 507 nm and 543 nm. Analysis of Ca^2+^ signals was performed in Fiji. ROIs were drawn around individual regions of membrane-localized fluorescence, with tissue movement corrected using the image stabilizer plugin. A recording from a unique lens was considered an individual replicate.

### Statistical analysis

For the iGluSnFR and eLACCO1.1 recordings, each cell was considered as an independent replicate. Pairwise comparions of the iGluSnFR and eLACCO1.1 recordings were made using the Mann–Whitney *U*-test. Dye loading data, where multiple comparisons were made, was analyzed with the Kruskal–Wallis ANOVA and *post hoc* Mann–Whitney *U*-tests. The Friedman 2-way ANOVA was used to analyze the Fluo-4 traces because multiple manipulations were performed on the same lens slice; thus, the lens slice was one factor and the pharmacological manipulation was the second factor. In this case, *post hoc* testing was performed via the Wilcoxon matched pairs signed rank test. All quantitative data are presented as box-and-whisker plots, where the line represents the median, the box is the interquartile range, and the whiskers represent the range, with all individual data points included. All calculations were performed on GraphPad Prism.

## Results

### Cx50 hemichannels possess the carbamylation motif and are CO_2_ sensitive

Alignment of the Cx50 sequence with that of Cx26 and Cx43, both of which are CO_2_ sensitive (Huckstepp et al., 2010a; Meigh et al., 2013; Dospinescu et al., 2026) revealed the presence of the carbamylation motif _140_KFRLEGT_146_ in Cx50. This sequence is analogous to the motifs _144_KVKMRGG_150_ in Cx43 and _125_KVRIEGS_131_ in Cx26. The carbamylation motif is within the cytoplasmic loop, which has proven to be notoriously difficult to resolve in cryoEM structures for all connexins (Maeda et al., 2009; Myers et al., 2018; Flores et al., 2020; Lee et al., 2020; Brotherton et al., 2022; Qi et al., 2023; Brotherton et al., 2024). Experimental Cx50 structures (e.g., 7JJP) show that K105 is in a similar orientation as K105 of Cx43 and R104 of Cx26. These latter residues form a salt bridge with the carbamylated Lys residue from the neighboring subunit. Understanding the location of K140 is more difficult. The AlphaFold3 prediction for the cytoplasmic loop and the position of K140 is of low confidence, and experimental structures do not resolve the entire cytoplasmic loop. However, some structures (e.g., 7JM9), while not resolving K140, do place R142 close to its location in the AlphaFold3 prediction, suggesting that the predicted positioning of K140 is likely to be approximately correct. Therefore, there is a reasonable probability that K140 is sufficiently close to K105 to form an inter-subunit salt bridge following carbamylation of K140.

As Cx50 appears to have the carbamylation motif, we tested its CO_2_ sensitivity by expressing a truncated variant of Cx50 in HeLa DH cells (see *Methods*). To assay hemichannel opening, we measured the possible efflux of either glutamate or lactate via Cx50 by co-expressing the genetically encoded sensors iGluSnFr or eLACCO1.1. We used these sensors rather than the more obvious choice of GRAB_ATP_ to assay ATP release (Butler and Dale, 2023), as we found in exploratory experiments that Cx50 hemichannels are not permeable to ATP (Lovatt et al., 2025).

**Figure 1:**
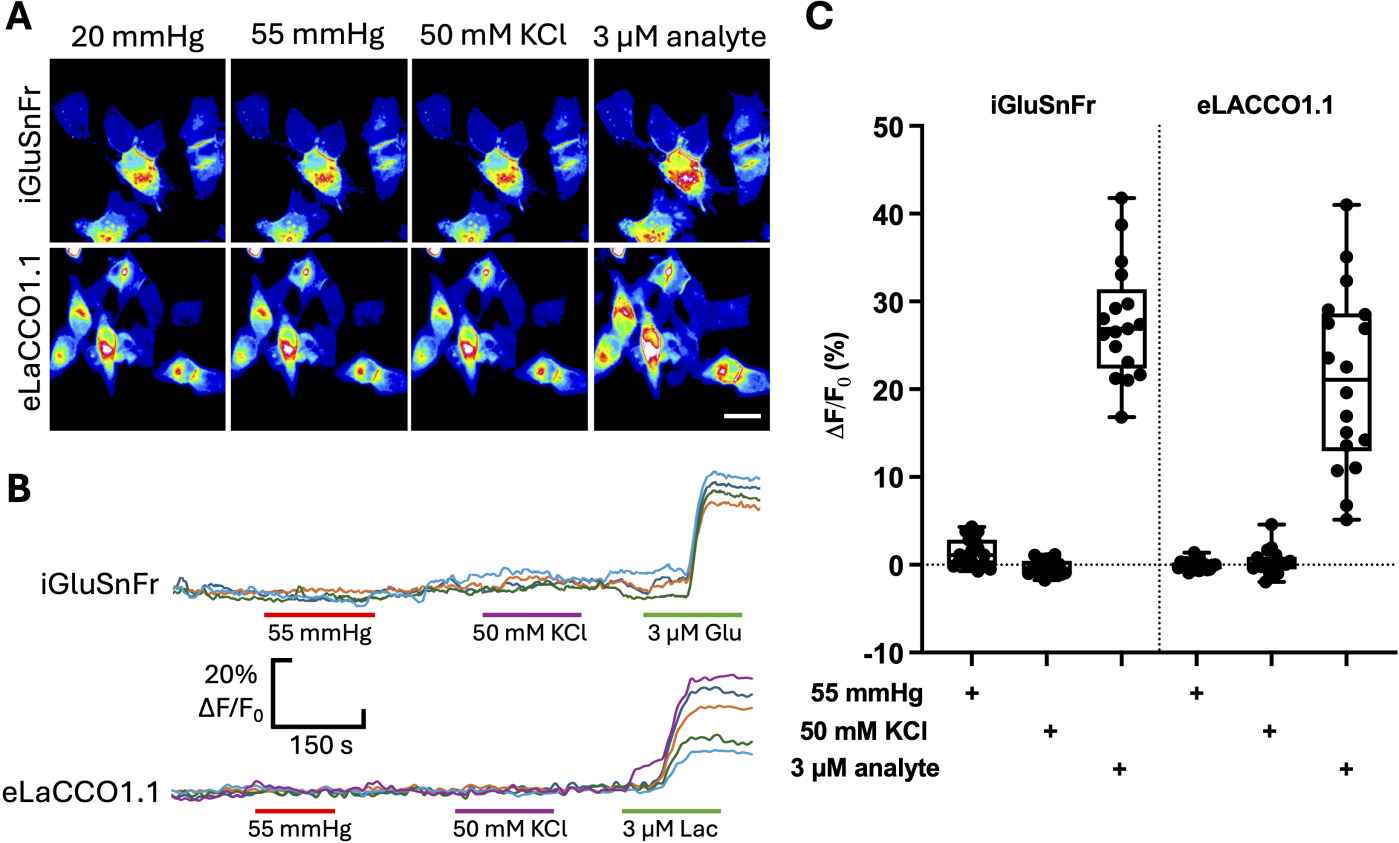
Parental HeLa DH cells do not release either glutamate or lactate in response to hypercapnia or depolarization. **(A)** Images of iGluSnFr or eLACCO1.1 fluorescence in HeLa cells. No change in fluorescence intensity was seen when the PCO_2_ of the aCSF was changed from 20 mmHg to 55 mmHg or when 50 mM KCl was applied. Scale bar 20 µm. **(B)** Traces showing the plot of fluorescence changes (ΔF/F_0_) versus time for the images in **(A)**. Red bar, application of 55 mmHg PCO_2_; purple bar, 50 mM KCl; green bar, application of the relevant analyte at 3 µM; all stimuli applied from a baseline of 20 mmHg PCO_2_. **(C)** Summary data showing that parental HeLa cells do not show fluorescence changes in response to CO_2_ or KCl. Data from three transfections each for iGluSnFr and eLACCO1.1.

**Figure 2:**
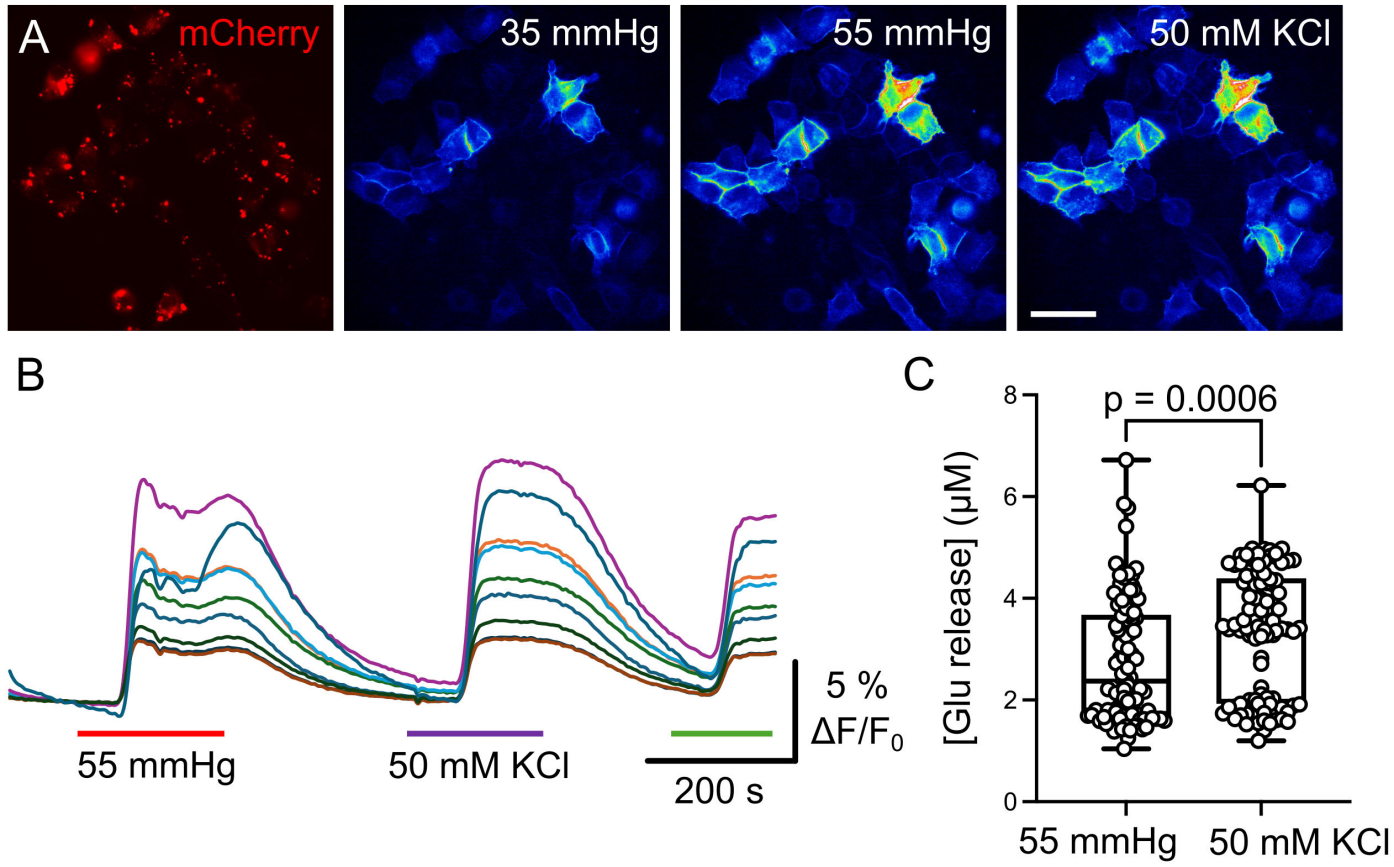
Full length Cx50 hemichannels are opened by changes in PCO_2_. **(A)** Images of Cx50 expression in HeLa cells (mCherry), and iGluSnFR fluorescence at 35 mmHg, 55 mmHg, and 50 mM KCl. Scale bar 20 µm. **(B)** Traces (each from a different ROI in the same field of view) showing change in iGluSnFr fluorescence over time. The recording was performed in aCSF with a PCO_2_ of 35 mmHg and the change to 55 mmHg (red bar), 50 mM KCl (purple bar), or 3 µM glu (green bar). **(C)** Summary data plotted as box and whisker plots to show CO_2_-evoked and KCl-evoked glutamate release. Each point is an individual ROI and data from five independent transfections.

**Figure 3:**
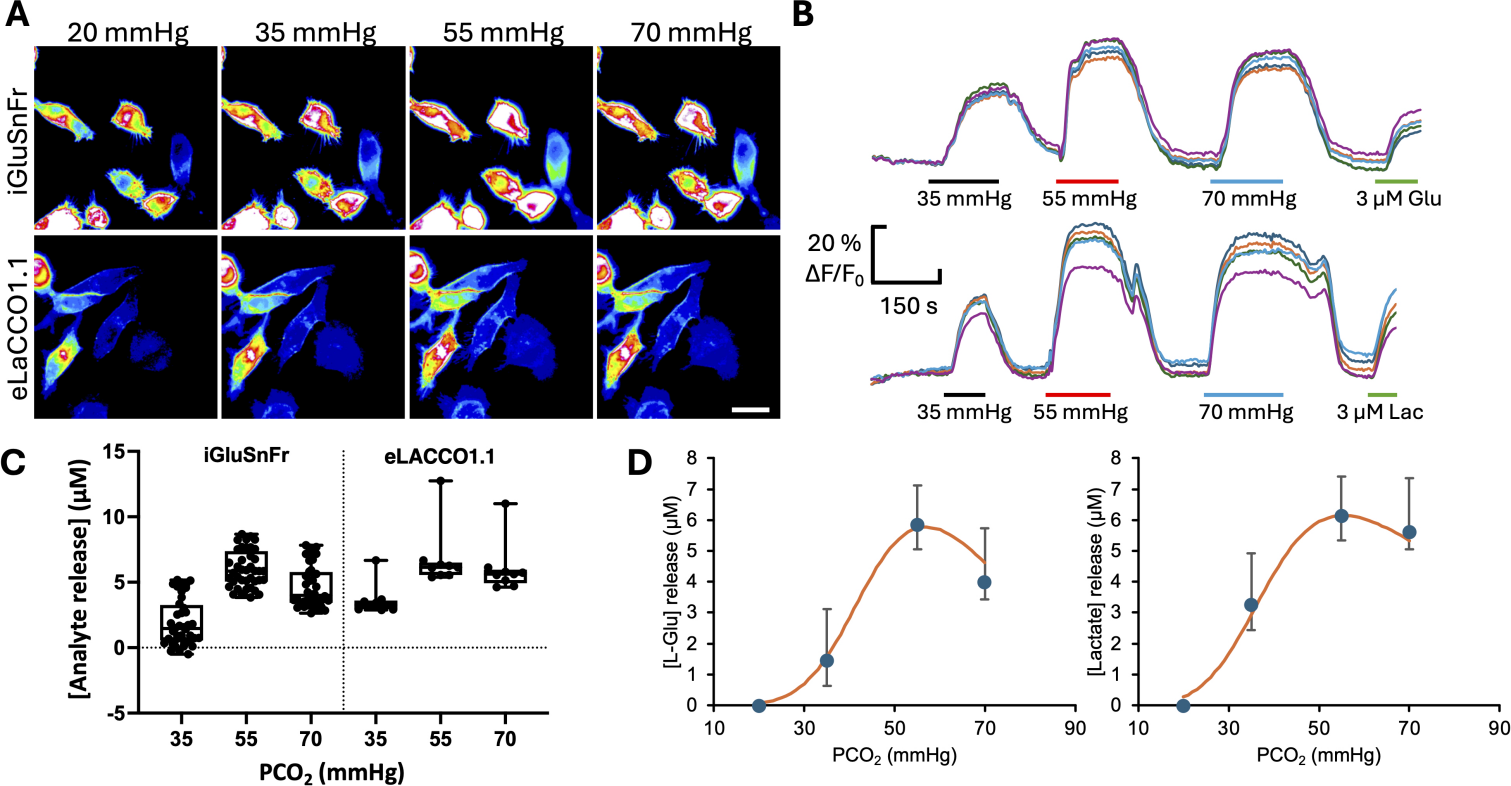
Connexin 50 truncated at the C-terminus is CO_2_ sensitive. **(A)** Images of iGluSnFr or eLACCO1.1 fluorescence in HeLa cells that express Cx50 to show the progressive larger changes in fluorescence intensity when the PCO_2_ of the aCSF was changed from 20 mmHg to 35 mmHg, 55 mmHg, and 70 mmHg. Scale bar 20 µm. **(B)** Traces showing the plot of fluorescence changes (ΔF/F_0_) for iGluSnFr (top) or eLACCO1.1 (bottom) versus time during application of 35 mmHg (black bar), 55 mmHg (red bar), 70 mmHg (light blue bar), and 3 µM analyte (green bar). **(C)** Summary data plotted as box and whisker plots showing CO_2_ evoked glutamate and lactate release (from four independent transfections for each analyte). **(D)** CO_2_-dependent release of glutamate and lactate from Cx50 expressing cells replotted as median with upper and lower quartiles. The continuous line is a modified Hill equation: [A] = Max. {(PCO_2_/K)^H^/(1 + (PCO_2_/K)^H^)}. {1/(1 + (PCO_2_/K_i_)^Hi^}. Where A is the analyte, K and H are the affinity and Hill coefficient of the channel for opening by CO_2_, K_i_ and H_i_ are the affinity and Hill coefficient for inhibition of the channel by CO_2_, and Max (µM) is the asymptotically maximum released concentration of the analytes to CO_2_. The parameters for the curves are: (L-Glu) H=6, K=46 mmHg, Max=10 µM, Ki=70 mmHg, Hi=5; (Lactate) H=5, K=42 mmHg, Max=11.5 µM, Ki=70 mmHg, Hi=3.

Parental HeLa cells that expressed only iGluSnFr or eLACCO1.1 did not exhibit fluorescence changes in response to hypercapnic and depolarizing stimuli (**Figure 1**). To test the possible CO_2_-sensitivity of Cx50 hemichannels, we next co-expressed this connexin with each of the genetically encoded sensors. When expressed in HeLa cells, full-length Cx50 showed a punctate expression pattern and gave glutamate release to both the 55 mmHg CO_2_ and KCl stimuli (**Figure 2**). There are many natural age-related truncations of the C-terminus of Cx50, and this may be a post-translational modification to reduce protein aggregation. To examine the CO_2_ sensitivity of Cx50 in more detail, we utilized a variant truncated at the C-terminus (Cx50^Δ277^, see *Methods*). The truncated variant showed similar punctate membrane expression to the full-length channel. Starting at a PCO_2_ of 20 mmHg, we successively switched to PCO_2–_35 mmHg, 55 mmHg, and 70 mmHg. We observed CO_2_-dependent fluorescence changes for both analytes (**Figure 3**). We therefore conclude that Cx50 hemichannels are CO_2_ sensitive and will permit CO_2_-dependent efflux of glutamate and lactate. Interestingly, significant release of glutamate and lactate was observed at a PCO_2_ of 35 mmHg. Maximal glutamate and lactate release was observed at a PCO_2_ of 55 mmHg, and some inhibition of release, relative to this maximum, was observed at a PCO_2_ of 70 mmHg (**Figures 3C, D**). Similar partial inhibition of hemichannel opening at high PCO_2_ has been observed in Cx43 (Dospinescu et al., 2026), but not in Cx26 or Cx32 (Huckstepp et al., 2010a; Dospinescu et al., 2019; Nijjar et al., 2025).

### The CO_2_-sensitivity of Cx50 depends on the carbamylation motif

**Figure 4:**
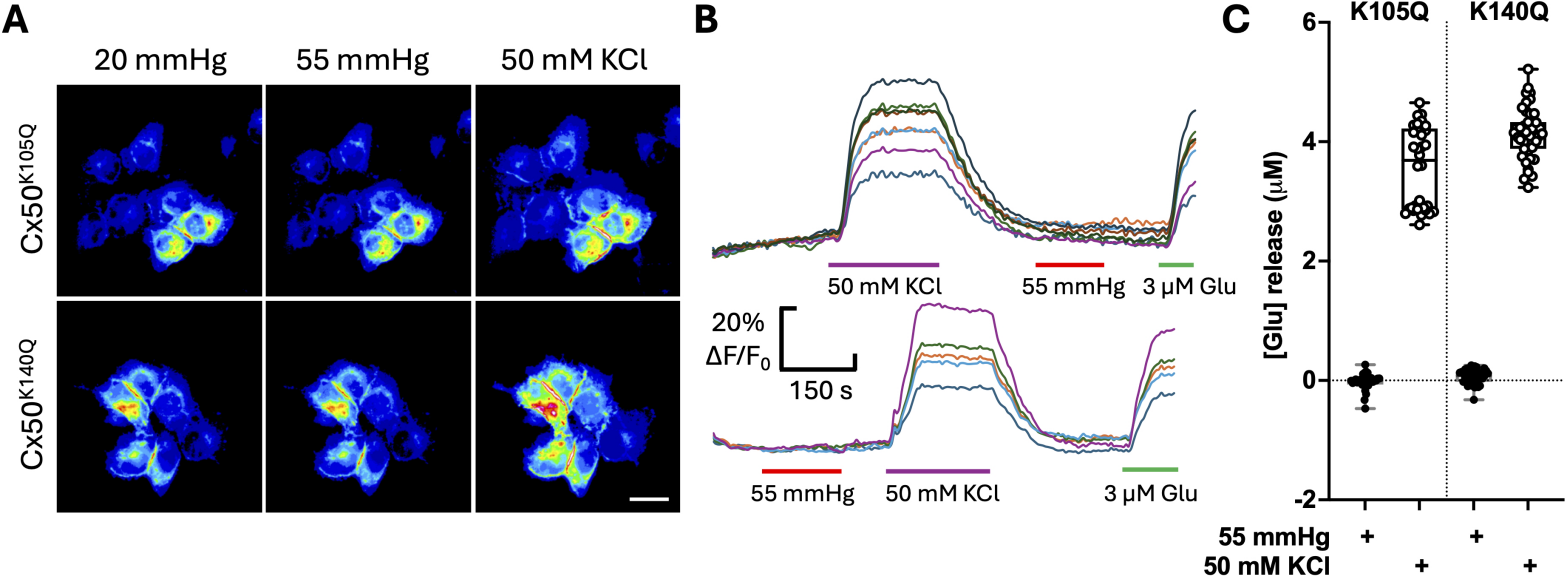
The CO_2_ sensitivity of truncated Cx50 depends on the carbamylation motif residues K140 and K105. **(A)** Images of iGluSnFr fluorescence showing that the mutations K105Q and K140Q abolish CO_2_-dependent glutamate release from HeLa cells expressing these mutant Cx50 hemichannels. A depolarizing stimulus (50 mM KCl) was still able to evoke glutamate release demonstrating that the mutant hemichannels remain voltage dependent and permeable to glutamate. Representative images from Video 1 and Video 2, respectively. Scale bar 20 µm. **(B)** Traces showing the plot of fluorescence changes (ΔF/F_0_) versus time for the images in **(A)**. Red bar, application of 55 mmHg PCO_2_; purple bar, 50 mM KCl; green bar 3 µM glutamate; all stimuli applied a baseline of 20 mmHg PCO_2_. **(C)** Summary data from four independent transfections for K105Q and five independent transfections of K140Q showing that these mutations abolish the CO_2_ evoked glutamate release.

We next tested whether the observed CO_2_ sensitivity of the truncated Cx50 hemichannels depended on K140 and K105 by individually mutating these residues to Gln. Gln cannot be carbamylated and, being neutral, will not be able to form a salt bridge with a carbamylated Lys residue. Application of PCO_2_ solutions at 55 mmHg to HeLa cells expressing either Cx50^K140Q^ or Cx50^K105Q^ was ineffective at evoking glutamate release (**Figure 4**; **Supplementary Videos 1** and **2**). As a positive control to demonstrate the presence of functional hemichannels, we used 50 mM KCl to depolarize the HeLa cells. This stimulus gave robust glutamate release, showing that the mutated channels remained voltage-sensitive and permeable to glutamate (**Figure 4**). We conclude that the action of CO_2_ in opening Cx50 hemichannels depends upon the carbamylation motif that we had previously identified in its structure.

### Some cataract forming mutations of Cx50 alter its CO_2_-sensitivity

**Figure 5:**
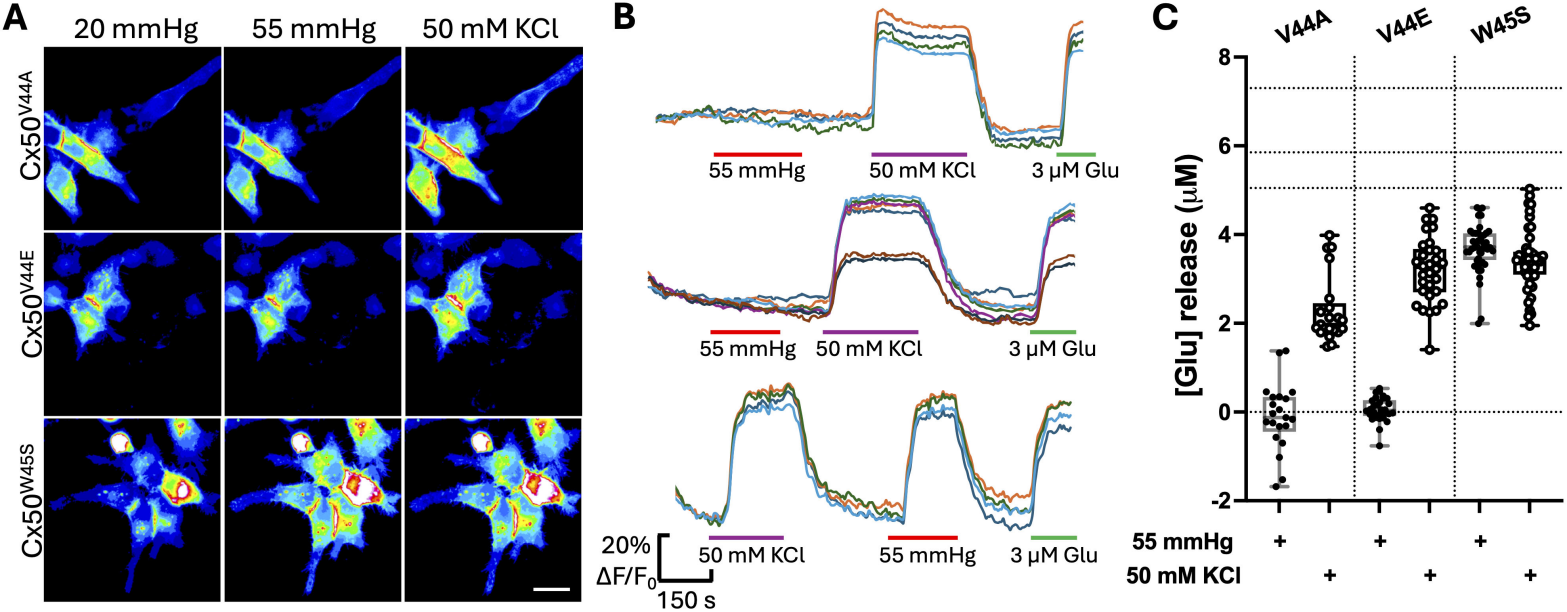
Some cataract forming mutations of Cx50 remove its CO_2_ sensitivity. **(A)** Images of iGluSnFr fluorescence showing that the mutations V44E and V44A but not W45S abolish CO_2_-dependent glutamate release from HeLa cells expressing these mutant Cx50 hemichannels. A depolarizing stimulus (50 mM KCl) is still able to evoke glutamate release demonstrating that the mutant hemichannels remain voltage dependent and permeable to glutamate. Scale bar 20 µm. **(B)** Traces showing the plot of fluorescence changes (ΔF/F_0_) versus time corresponding to the images in **(A)**. Red bar, application of 55 mmHg PCO_2_; purple bar 50 mM KCl; green bar, 3 µM glutamate; all stimuli applied from a baseline of 20 mmHg PCO_2_. **(C)** Summary data for: V44A, four independent transfections; V44E, four independent transfections; and W45S, five independent transfections. The dotted lines at top of y-axis show the values for the median CO_2_-evoked glutamate release with lower and upper quartiles for Cx50^WT^. Cx50^V44A^ and Cx50^V44E^ are insensitive to CO_2_, while Cx50^W45S^ releases significantly less glutamate to 55 mmHg than the wildtype channel (MW Test, p <0.0001).

Cx50 plays a key role in the physiology of the lens, and certain mutations cause cataract formation (Shiels et al., 1998; Berthoud and Ngezahayo, 2017; Berthoud et al., 2020; Shiels and Hejtmancik, 2021; Shi et al., 2022). We have examined whether three of these mutations, V44A, V44E, and W45S, alter the CO_2_ sensitivity of the truncated channel (**Figure 5**). We found that both mutations of V44 abolished the CO_2_ sensitivity of Cx50 hemichannels, but the mutated channels remained voltage-sensitive and permeable to glutamate. However, Cx50^W45S^ hemichannels remained CO_2_-sensitive, but were significantly less CO_2_-sensitive than Cx50^WT^ (p <0.0001, **Figure 5**). Our results raise the possibility that loss or reduction of the CO_2_ sensitivity of Cx50 could be a contributing cause to cataract formation.

**Figure 6:**
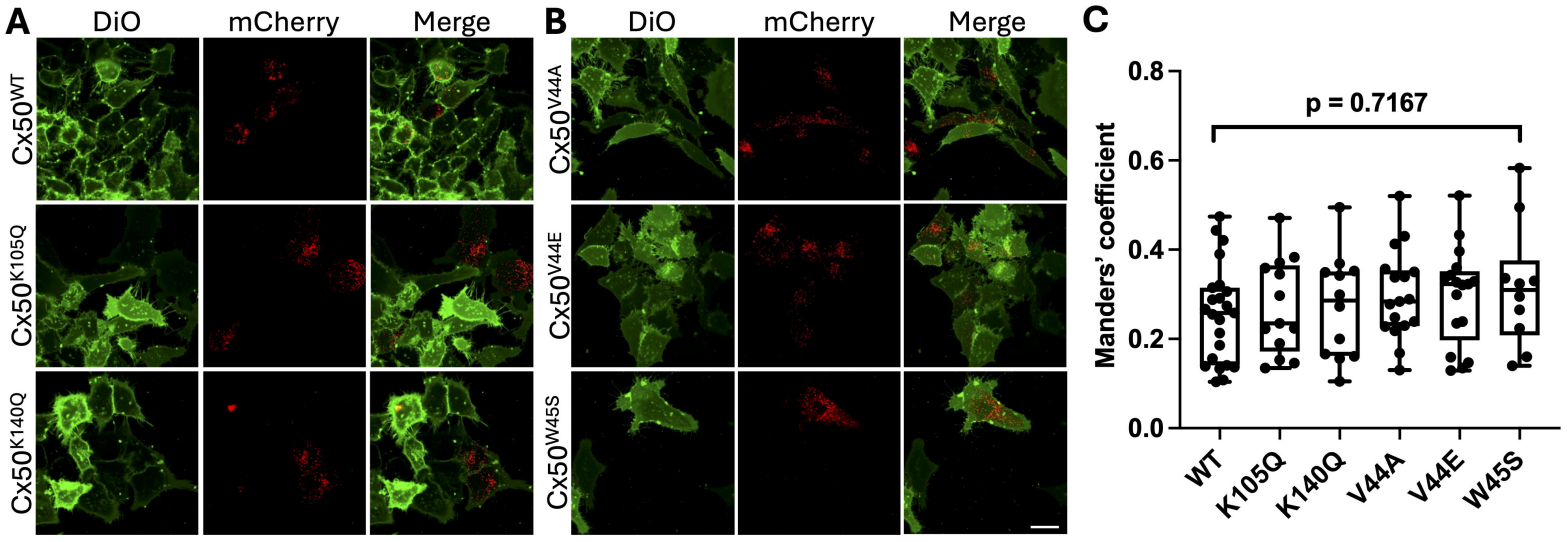
Carbamylation motif mutations and cataract forming mutations do not alter expression of truncated Cx50 at the plasma membrane. **(A, B)** Confocal images (single optical plane) showing membrane staining with DiO (green) and the mCherry C-terminal tag on the Cx50 variants (red). Scale bar 20 µm. **(C)** Measurement of the Manders’ coefficient (the proportion of DiO fluorescence that colocalizes with mCherry) for each Cx50 variant. These coefficients do not differ between the wild type and the mutants (Kruskal–Wallis ANOVA, p = 0.7167). Data from three independent transfections for each variant.

To determine whether the results of the mutations could be explained by altered membrane localization, we stained the plasma membrane with DiO and examined the colocalization of DiO with the mCherry tag on Cx50 for the WT channel and all mutated variants using confocal microscopy (**Figure 6**). This analysis showed that the Manders’ coefficients were the same for all Cx50 variants. Thus, the mutations did not alter membrane localization of Cx50 within the resolution limits of light microscopy.

### The lens is CO_2_ sensitive

Cx50 is expressed in lens fiber cells, and along with Cx46, is the major connexin subtype in this tissue. Given that Cx50 is CO_2_-sensitive, we might expect that this would endow the lens itself with CO_2_ sensitivity. We therefore used a dye-loading assay to test this (Huckstepp et al., 2010b). We exposed acutely isolated whole lenses to a membrane-impermeant dye, FITC, at different levels of PCO_2_. Were Cx50 in the lens to open, it should permit the entry of FITC into the fiber cells.

**Figure 7:**
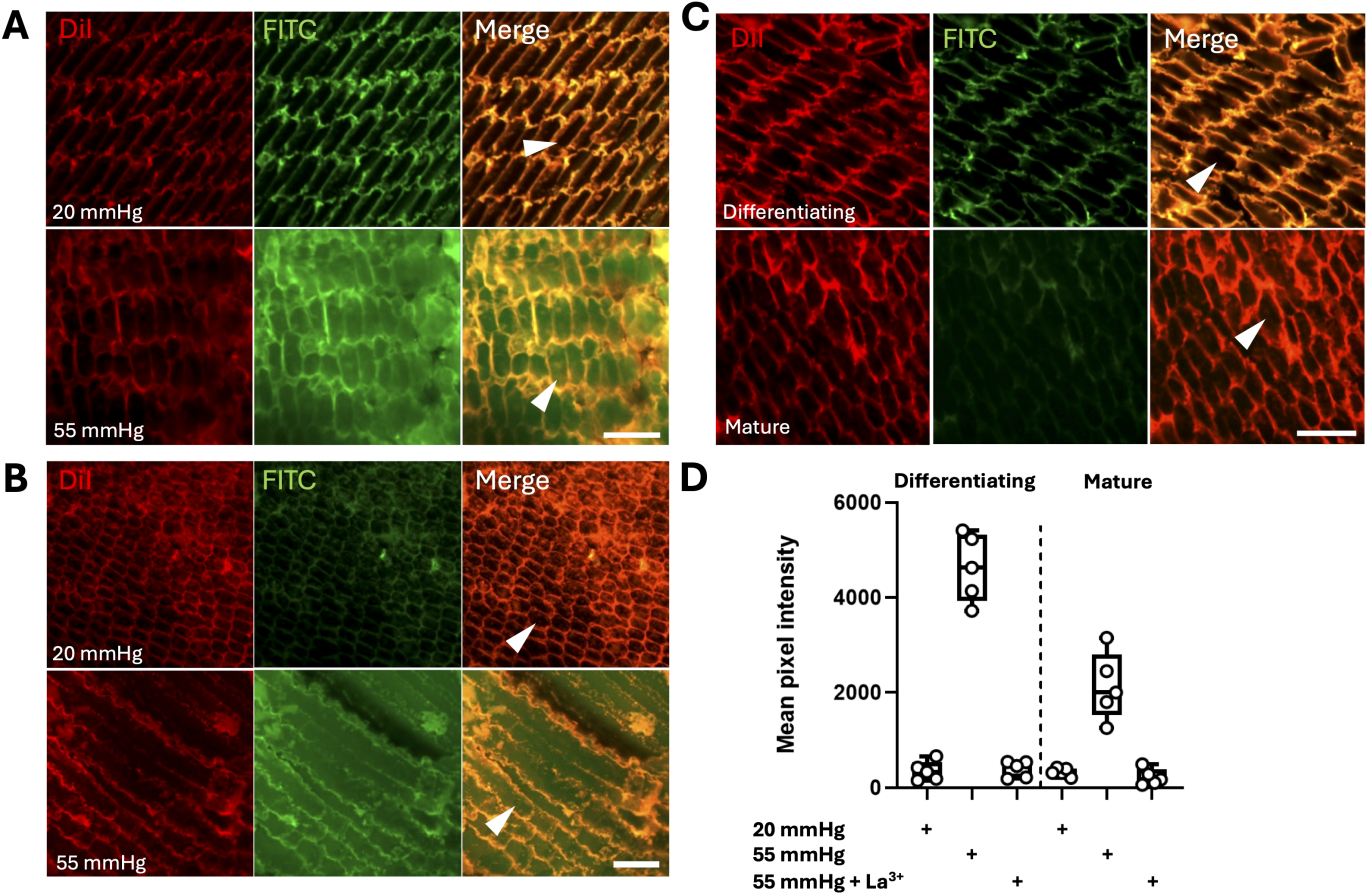
Hemichannel-mediated CO_2_-dependent dye loading into lens fiber cells. Differentiating **(A)** and mature **(B)** fiber cells load with the membrane impermeant dye (FITC, green) at a PCO_2_ of 55 mmHg but not at 20 mmHg. The sections of lens tissue have been stained with DiI (red) to label the membranes. Some FITC is present on the outside of the membrane in both conditions, but FITC is only present within the cytosol (arrow heads) at the higher level of PCO_2_. **(C)** Dye loading at 55 mmHg PCO_2_ is completely blocked by the hemichannel inhibitor, La^3+^ (200 µM) -no FITC is present in the cytosol of either differentiating or mature lens fiber cells (arrow heads). **(D)** Summary graph showing for five lens preparations under each condition, the amount of FITC loading in fiber cells. Each individual data point represents the mean pixel intensity of at least 10 cells from a unique lens (n = 5 lens). Kruskal–Wallis ANOVA: Differentiating fibers, p <0.0001, Mature fibers, p <0.0001. *Post hoc* Mann–Whitney U tests: Differentiating fibers; 20 mmHg vs 55 mmHg, p = 0.0056, 20 mmHg vs 55 mmHg + La^3+^, p = 0.7682, Mature fibers; 20 mmHg vs 55 mmHg, p = 0.0005, 20 mmHg vs 55 mmHg + La^3+^, p = 0.9492. Scale bars 25 µm **(A)**, 40 µm **(B)**, 25 µm **(C)**.

Differentiating and mature lens fiber cells were identified based on their position, with the former located at the center of the lens and the latter at the periphery, as determined by an initial low-power image used to visualize the entire section. At a PCO_2_ of 20 mmHg (when Cx50 hemichannels would be shut), we did not observe FITC loading in either differentiating or mature fiber cell types (**Figure 7**). FITC was frequently observed to accumulate between the plasma membranes, without entering cells. Trapping of Lucifer Yellow has also been observed between lens fiber cells (Suzuki-Kerr et al., 2022). By contrast, at a PCO_2_ of 55 mmHg, FITC fluorescence was observed in the cytosol of both differentiating and mature fiber cells (**Figure 7**).

To test whether the FITC entry might occur via a hemichannel, we used the blocker La^3+^ which blocks many types of hemichannels (connexin and pannexin) but does not block many types of voltage-gated Na^+^, K^+^ or Ca^2+^ channels. La^3+^ completely blocked FITC entry during exposure to a PCO_2_ of 55 mmHg (**Figure 7**), indicating that this occurred via a hemichannel. As Cx50 hemichannels are the only known CO_2_-sensitive channels with large pores that could plausibly permit the entry of FITC, it is very likely that these hemichannels are a conduit for FITC entry. Cx46 hemichannels, if CO_2_-sensitive, might also play a role in FITC entry (see *Discussion*).

### Lens fiber cells exhibit CO_2_ dependent Ca^2+^ entry

**Figure 8:**
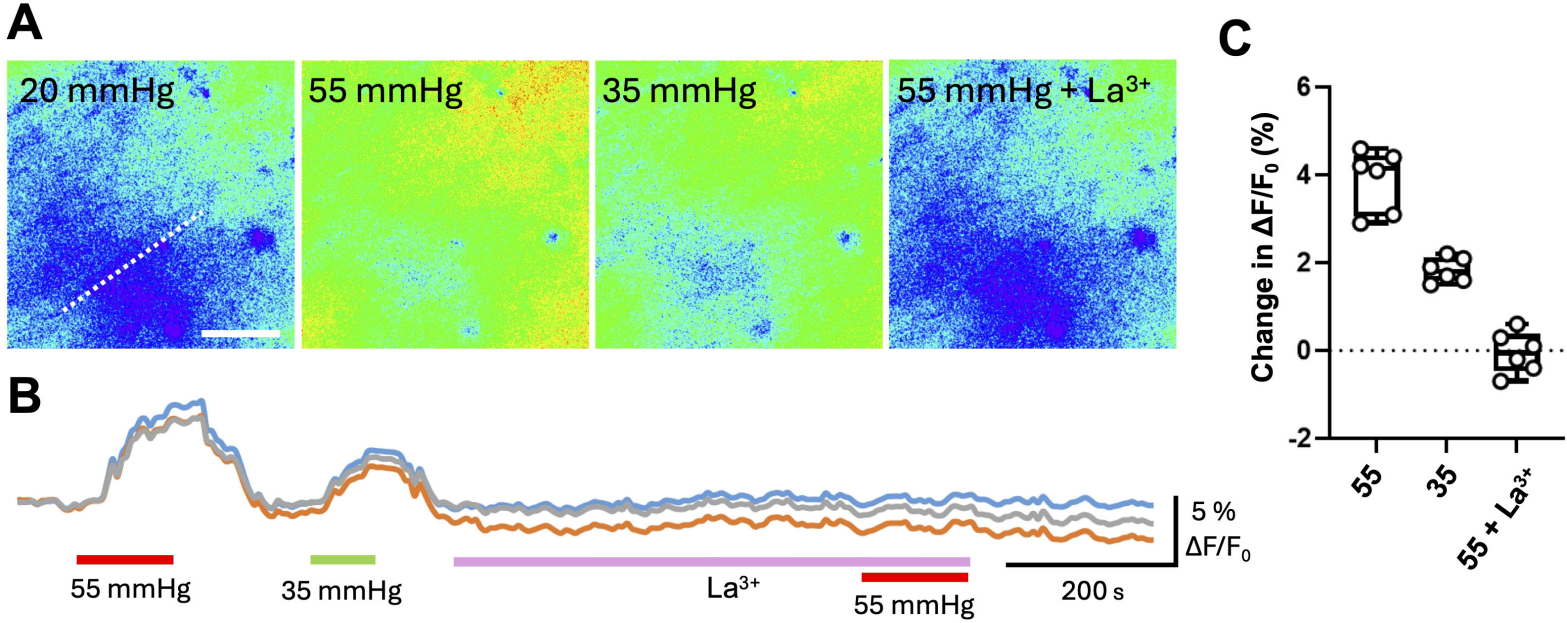
Hemichannel mediated CO_2_ dependent Ca^2+^ influx into lens fiber cells. **(A)** Sample images of Fluo-4 loaded lens slices. The dotted line is parallel to the long axis of the fiber cells in the slice. A large increase of fluorescence was seen at 55 mmHg and 35 mmHg PCO_2_ compared to the baseline (20 mmHg). The increase in fluorescence evoked by 55 mmHg PCO_2_ was blocked by 200 µM La^3+^. Scale bar, 50 µm. **(B)** Quantification of the Fluo-4 fluorescence changes evoked by 55 mmHg and 35 mmHg and the blocking effect of La^3+^ on the increase in fluorescence. **(C)** Summary data from six different lens slices showing the changed in normalized fluorescence under the three conditions. Friedman 2-way ANOVA: p = 0.0278. Wilcoxon matched-pairs signed rank test: 55 mmHg vs 35 mmHg PCO_2_, p = 0.0313.

Given that lens microcirculation depends on ion influxes in lens fiber cells, we sought to gain evidence for CO_2_-dependent ion entry into lens fiber cells. As it is much easier to measure intracellular Ca^2+^ than Na^+^, and because connexin hemichannels are permeable to both Na^+^ and Ca^2+^ ions, we loaded acute lens slices with Fluo-4 to test whether changes in PCO_2_ might trigger a Ca^2+^ influx. Using a baseline PCO_2_ of 20 mmHg, we demonstrated that increases to either 35 mmHg or 55 mmHg triggered a very substantial influx of Ca^2+^ that could be blocked by pre-treatment with La^3+^ (**Figure 8**). This indicates that the opening of a CO_2_-sensitive hemichannel, most likely Cx50, provides a Ca^2+^ influx. Crucially, the CO_2_ sensitivity of this influx matches the dose–response relationship of Cx50 that we established in **Figure 2** and shows that even at the likely resting PCO_2_ of the aqueous humor, Cx50 hemichannels in lens fiber cells will be partially open.

### Lens fiber cells respond to glutamate via NMDA receptors

A curious feature of Cx50 hemichannels is that they are impermeable to ATP, but permeable to both glutamate and lactate (Lovatt et al., 2025). This led us to question whether glutamate efflux through the opening of Cx50 hemichannels might be physiologically important in the lens. There are several reports of AMPA and NMDA receptors being expressed on lens fiber cells (Farooq et al., 2012; Bhattacharyya et al., 2014; Frederikse et al., 2016), but the source of the activating glutamate and the receptors’ roles in the physiology of the lens remain unknown.

**Figure 9:**
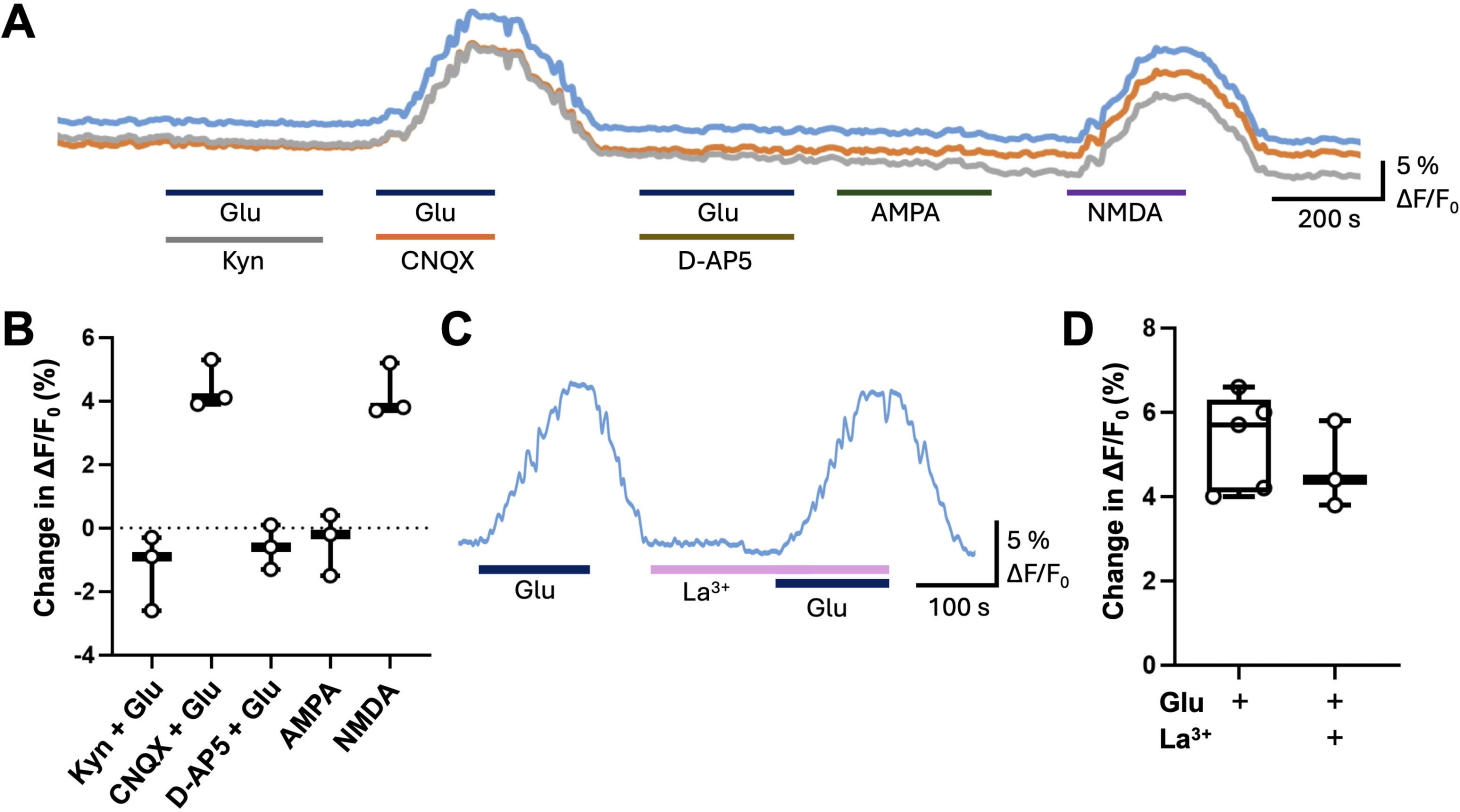
Glutamate evoked Ca^2+^ signals in lens fiber cells are mediated by NMDA receptors. **(A)** Traces of normalized Fluo-4 fluorescent showing the response to 200 µM L-glutamate in presence of 1 mM kynurenic acid, 10 µM CNQX or 100 µM D-AP5, and the selective ionotropic glutamate receptor agonists AMPA (5 µM) and NMDA (60 µM). **(B)** Summary data for the responses to glutamate receptor agonists for n = 3 lens slices. Friedman 2-way ANOVA: p = 0.0053. **(C, D)** The responses to glutamate (as measured by Fluo-4 fluorescence) are not dependent on hemichannel activation and are unaffected by 200 µM La^3+^, n = 5 lens slices for Glu and n = 3 lens slices for Glu + La^3+^.

We therefore tested whether glutamate might evoke Ca^2+^ signals in lens fiber cells. Using Fluo-4-loaded acute lens slices, we found that glutamate evoked robust increases in intracellular Ca^2+^. These could be blocked by the general glutamate-receptor antagonist kynurenic acid and the NMDA receptor-selective antagonist D-AP5, but not by the AMPA receptor-selective antagonist, CNQX (**Figure 9**). The glutamate responses were thus mediated via NMDA receptors. Note that we cannot exclude a role for the AMPA receptor in mediating ion influx, but as the AMPA receptor in the lens undergoes post-transcriptional Q/R editing (Farooq et al., 2012) it is impermeable to Ca^2+^ and we would therefore not be able to observe any contribution from this receptor using Fluo-4 imaging. The glutamate responses were not blocked by La^3+^, indicating that they were independent of any hemichannel activity (**Figure 9**).

### CO_2_-evoked glutamate release contributes to the CO_2_ dependent Ca^2+^ influx in lens fiber cells

**Figure 10:**
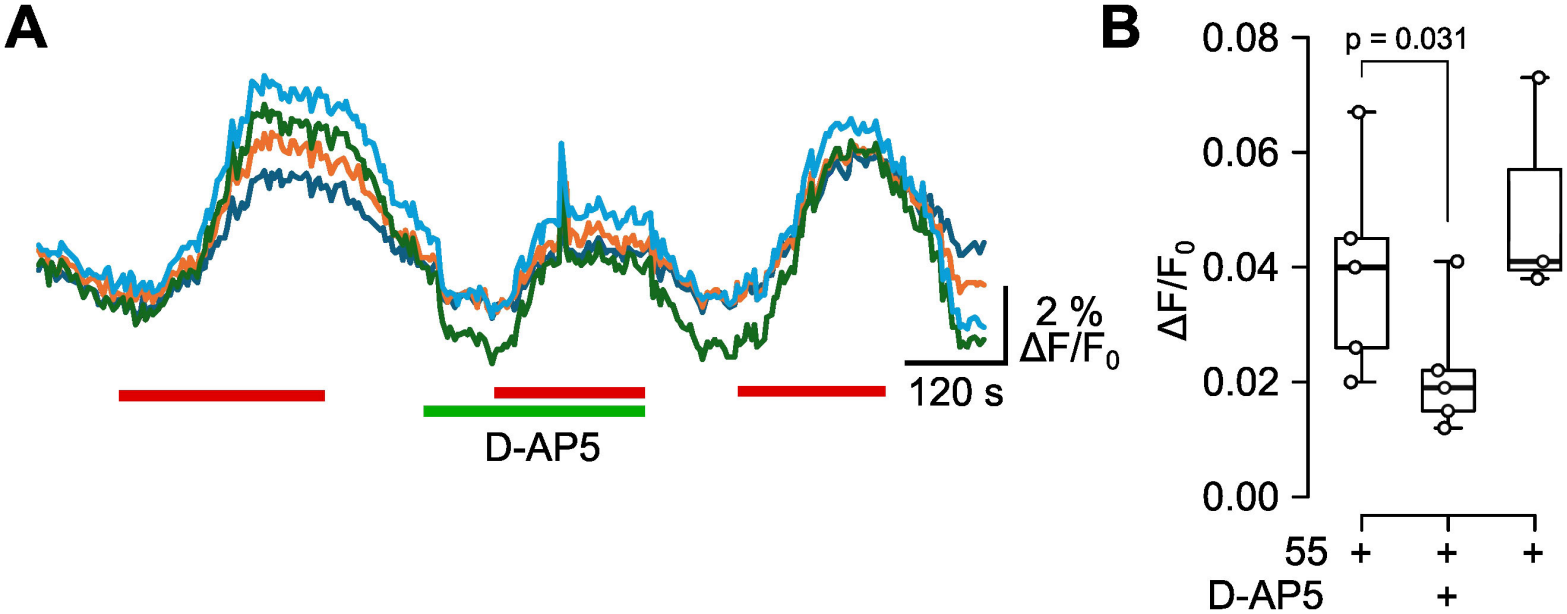
Activation of NMDA receptors during a CO_2_ challenge partially contributes to the elevation of intracellular Ca^2+^ in lens fiber cells. **(A)** Traces from ROIs on a Fluo-4 loaded lens slice showing responses to 55 mmHg PCO_2_ (red bars) before, during and after application of 100 µM D-AP5 (green bar). **(B)** Summary graph showing effect of 100 µM D-AP5 on change in intracellular Ca^2+^ evoked by 55 mmHg PCO_2_; n = 5 lens for 55 mmHg and 55 mmHg + D-AP5, n = 3 lens for wash after 55 mmHg + D-AP5. Wilcoxon matched pairs signed rank test, p = 0.031, 55 mmHg vs 55 mmHg + D-AP5.

Given that Cx50 hemichannels permit the CO_2_-dependent release of glutamate, we asked whether the Ca^2+^ influx triggered by a PCO_2_ of 55 mmHg might be at least partially mediated via NMDA receptors. We therefore loaded lens fiber cells with Fluo-4 and first measured the change in intracellular Ca^2+^ evoked by the PCO_2_ challenge and then applied D-AP5 prior to the PCO_2_ challenge to see whether NMDA receptor antagonism might reduce the Ca^2+^ signal. We found that D-AP5 reversibly reduced the Ca^2+^ influx by roughly half (**Figure 10**). This implies that, during elevated PCO_2_, the opening of Cx50 hemichannels permits not just the influx of Ca^2+^ but also the efflux of glutamate, which can then activate NMDA receptors to provide an additional Ca^2+^ influx. The lens fiber cells are thus a source of the glutamate that activates the receptors on their surfaces.

## Discussion

Cx50 is the fifth connexin, after Cx26, Cx30, Cx32, and Cx43, that is now known to be CO_2_-sensitive. Significantly, it is the second alpha connexin alongside Cx43 documented to have this property. All five connexins share a carbamylation motif that was originally identified in Cx26 and closely related beta connexins. The mechanism of CO_2_-dependent hemichannel opening in Cx43 involves four Lys residues: K105, K109, K144, and K234 (Dospinescu et al., 2026). Cx50 lacks K109 (this residue is an Arg), and it is interesting that mutation of either K105 or K140 in Cx50 is sufficient to abrogate CO2 sensitivity. In Cx43, at least two Lys residues must be mutated to abolish CO2 sensitivity (Dospinescu et al., 2026). Thus, the mechanism of CO2 sensitivity in Cx50 is somewhat simpler than in the related Cx43 and more like that proposed for Cx26 (Meigh et al., 2013; Brotherton et al., 2022, 2024).

### Could Cx50 hemichannels act as homeostatic regulators of lens microcirculation?

The properties of Cx50 hemichannels appear to be matched to the physiology of the lens. Firstly, Cx50 hemichannels are significantly open at a PCO_2_ of 35 mmHg. The PCO_2_ of the aqueous humor is generally a little lower than that measured in plasma (Salit, 1930; Krupin et al., 1980; Sharma et al., 1983) and is in the range of 35 mmHg–40 mmHg. According to our data, therefore, Cx50 hemichannels would be expected to be partially open and provide a continual source of Na^+^ ions to power the microcirculation. Secondly, the degree of opening of Cx50 will be proportional to PCO_2_ over the relevant physiological range. We propose the hypothesis that CO_2_, acting via Cx50, could mediate homeostatic control of the microcirculation. Under this hypothesis, were PCO_2_ to rise above its normal resting level, it would imply insufficient microcirculation and waste removal. As a consequence of the increased PCO_2_, Cx50 hemichannels would open more and allow a greater Na^+^ influx, leading to faster microcirculation and a corrective adaptive response. Conversely, if PCO_2_ were to fall below the normal resting level, it would imply that the microcirculation was too fast. Cx50 hemichannels would close in response to the lowered PCO_2_, reducing the Na^+^ influx and hence the rate of microcirculation, once again leading to a corrective adaptive response.

Thirdly, Cx50 hemichannels are permeable to lactate (Lovatt et al., 2025). To maintain the transparency of the lens, as lens fiber cells mature, they lose their organelles, including mitochondria (Bassnett, 2002). This means that they become dependent on ATP supply from more metabolically active cells via gap junctions and from glycolysis. Lactate is the end product of glycolysis, and thus the permeability of Cx50 to lactate potentially provides an effective way of allowing the efflux from the fiber cells of this end product.

### Supporting evidence for the proposed homeostatic role of Cx50 hemichannels

We have shown that the lens is indeed CO_2_-sensitive with characteristics consistent with the properties of Cx50 hemichannels. Lens fiber cells load with FITC in a CO_2_-sensitive manner, and we recorded a CO_2_-dependent Ca^2+^ influx that is likely through Cx50, as it matched the CO_2_ dose–response relationship of the Cx50 hemichannel and, like the FITC loading, could be blocked by La^3+^. While this does not definitively demonstrate that the lens’s CO_2_ sensitivity depends on Cx50, the balance of probabilities makes this likely.

The human gene for Cx46 lacks the carbamylation motif and is not CO_2_-sensitive (Lovatt et al., 2025). However, rodent Cx46 does possess the carbamylation motif, is potentially CO_2_-sensitive and might contribute to the overall sensitivity of lens to CO_2_. Unlike Cx50, Cx46 is not permeable to glutamate (Lovatt et al., 2025). Therefore, even if CO_2_-sensitive, Cx46 could not contribute to the CO_2_-evoked glutamate release observed in the lens. Interestingly, genetic knock-in of Cx46 into the Cx50 locus is unable to fully rescue the loss of Cx50 functionality (Wang et al., 2017) showing that Cx50 must have unique functional properties necessary for a healthy lens.

Our evidence gives some support for Cx50 hemichannels acting as homeostatic regulators of the lens microcirculation. Firstly, there was an influx of Ca^2+^ into fiber cells at a PCO_2_ of 35 mmHg. This supports our speculation that Cx50 hemichannels in lens fiber cells may be partially open at the resting PCO_2_ typical of the aqueous humor. Secondly, the Ca^2+^ influx increases with increasing PCO_2_, supporting the second component of our hypothesis. Thirdly, our evidence ties together a curious property of the Cx50 hemichannel, its permeability to glutamate, with the existence of ionotropic glutamate receptors on lens fiber cells. We found that the NMDA receptor antagonist D-AP5 partially reduced the CO_2_-induced Ca^2+^ influx into the fiber cells. This is most simply explained by the opening of Cx50 hemichannels not only allowing the influx of Ca^2+^ but the efflux of glutamate, which can then activate NMDA receptors to provide an additional component of Ca^2+^ influx. We note that both Cx50 hemichannels and NMDA receptors are highly permeable to Na^+^ and thus will act as sources (presumably via gap junction coupling) of Na^+^ for the Na^+^/K^+^ ATPases in lens epithelial cells. Furthermore, glutamate released through Cx50 hemichannels will most probably also activate the AMPA receptors present on the fiber cells, even though we cannot directly observe this using Ca^2+^ imaging.

While many investigators have queried the possible identity of the channel that provides the Na^+^ inward leak current to power microcirculation, our data suggest that this Na^+^ influx may originate from multiple channels: Cx50 as the trigger, with downstream activation via glutamate release activating the NMDA receptor, and very likely the AMPA receptor, thereby providing additional sources of Na^+^ influx. There is also evidence that Cx46 hemichannels could contribute to a Na^+^ leak current in lens fiber cells (Ebihara et al., 2014). It is important to note that we have not directly measured lens microcirculation and that definitive testing of our hypothesis requires demonstration that the rate of microcirculation in the lens can be altered in a CO_2_-dependent manner that is consistent with the properties of Cx50 hemichannels and the involvement of glutamate receptors.

### Pathological mutations of Cx50 alter its CO_2_ sensitivity

If Cx50 hemichannels really are key regulators of the microcirculation in the lens, at least some cataract-forming mutations of Cx50 should alter its CO_2_ sensitivity. We have previously shown that a subset of pathological mutations of Cx26, Cx32, and Cx43 that respectively cause keratitis ichthyosis deafness syndrome (KIDS), X-linked Charcot-Marie-Tooth disease (CMTX) and oculodentodigital dysplasia (ODDD) abolish the CO_2_ sensitivity of these hemichannels (Meigh et al., 2014; de Wolf et al., 2016; Cook et al., 2019; Butler and Dale, 2023; Dospinescu et al., 2026). In Cx26, mutations that cause KIDS such as A88V, A40V, and N14K occur at residues near points of flexibility in the molecule. Substitution of a bulkier residue may impede this flexibility and prevent the ability of the protein to respond to CO_2_ (Brotherton et al., 2022, 2024).

Our observation that some cataract-forming mutations of Cx50 also modify its CO_2_ sensitivity therefore fits the pattern of the effects of pathological mutations observed in other connexins. We suggest by analogy that the modification of CO_2_ sensitivity of Cx50 may arise from alterations to the flexibility of the molecule caused by these mutations. Our observation is consistent with, but does not substantiate, the hypothesis that the loss of CO_2_ sensitivity of Cx50 contributes to cataract formation. As Cx50^W45S^ retains reduced CO_2_-sensitivity yet also causes cataracts, loss of other properties of Cx50 must also contribute to cataract formation. One might imagine that any mutations that alter the permeability of Cx50 hemichannels to glutamate might also cause cataracts if our hypothesis is correct. Clearly, examination of further cataract mutations will be required to understand any potential role that loss of CO_2_ sensitivity might have in the etiology of cataract formation. In addition, testing whether mutations that directly target CO_2_ sensitivity (e.g., K140Q) are sufficient to trigger cataract formation would be a more rigorous test of this hypothesis.

## Data Availability

The original contributions presented in the study are included in the article/**Supplementary Material**. Further inquiries can be directed to the corresponding author.

## Supplementary material

Supplementary Video 1 Cx50^K105Q^ does not respond to CO_2_ but remains voltage sensitive.

Supplementary Video 2 Cx50^K140Q^ does not respond to CO_2_ but remains voltage sensitive.

Supplementary data file Raw data for all graphs in the figures.
